# The ethics of human volunteer studies involving experimental exposure to pesticides: unanswered dilemmas

**DOI:** 10.1186/1476-069X-9-50

**Published:** 2010-08-18

**Authors:** Leslie London, David Coggon, Angelo Moretto, Peter Westerholm, Martin F Wilks, Claudio Colosio

**Affiliations:** 1 Centre for Occupational and Environmental Health Research, School of Public Health and Family Medicine, University of Cape Town, Cape Town, South Africa; 2 MRC Epidemiology Resource Centre, University of Southampton, Southampton, UK; 3 Department of Occupational and Environmental Health, Università degli Studi di Milano, Milan, Italy; 4 Department of Medical Sciences, Uppsala University, Akademiska sjukhuset, Uppsala, Sweden; 5 Swiss Centre for Applied Human Toxicology, University of Basel, Klingelbergstrasse 61, Basel, Switzerland; 6 Department of Occupational and Environmental Health, Università degli Studi di Milano, Italy; 7 International Centre for Pesticides and Health Risk Prevention, "Luigi Sacco" Hospital, Milan, Italy; 8 International Centre for Rural Health, San Paolo Hospital, Via di Rudini 8, 20124 Milan, Italy

## Abstract

The controversy about the use of data from human volunteer studies involving experimental exposure to pesticides as part of regulatory risk assessment has been widely discussed, but the complex and interrelated scientific and ethical issues remain largely unresolved. This discussion paper, generated by authors who comprised a workgroup of the ICOH Scientific Committee on Rural Health, reviews the use of human experimental studies in regulatory risk assessment for pesticides with a view to advancing the debate as to when, if ever, such studies might be ethically justifiable. The discussion is based on three elements: (a) a review of discussion papers on the topic of human testing of pesticides and the positions adopted by regulatory agencies in developed countries; (b) an analysis of published and unpublished studies involving human testing with pesticides, both in the peer-reviewed literature and in the JMPR database; and (c) application of an ethical analysis to the problem. The paper identifies areas of agreement which include general principles that may provide a starting point on which to base criteria for judgements as to the ethical acceptability of such studies. However, the paper also highlights ongoing unresolved differences of opinion inherent in ethical analysis of contentious issues, which we propose should form a starting point for further debate and the development of guidelines to achieve better resolution of this matter.

## Introduction

In recent years, there has been considerable controversy about the scientific value and ethical acceptability of studies involving experimental exposure of human volunteers to low doses of pesticides [1-12]. Although such studies have been conducted for many decades, albeit on a limited scale, the controversy around their use has been prompted particularly by more recent debates on the use of data from human volunteer studies to inform regulatory risk assessment [13,14].

This issue emerged in the public domain as a result of changes in the regulatory framework in the United States through the Food Quality Protection Act [15] and the subsequent submission of data from experimental volunteer studies to support the setting of toxicological reference values for certain pesticides [16-20]. The subsequent debate has led to a critical examination of the use of human data in general, and its use in pesticide regulation in both the USA and the European Union and in the deliberations of the FAO/WHO Joint Meeting on Pesticide Residues (JMPR).

In contrast to many other chemicals, regulatory assessment of risks to human health from pesticides is typically based on a wide set of studies in vitro and in vivo in animals, sometimes supplemented by observational studies (primarily epidemiological investigations though sometimes case reports and case series may be used) in humans. Animal studies examine both kinetics (absorption, distribution, metabolism and excretion, ADME studies) and toxic effects. A final outcome of many toxicity studies is the identification of No Observed Adverse Effect Levels (NOAELs) and Lowest Observed Adverse Effect Levels (LOAELs), which are used to derive various toxicological reference values. It is customary to include uncertainty factors (also known as safety or assessment factors) to account for individual variability and uncertainties in extrapolation to humans, and sometimes also to allow for limitations of experimental design. A default value of 100 is frequently used, which consists of two ten-fold factors: one to account for possible interspecies differences and one to reflect human inter-individual variability. The resulting reference value represents the maximum exposure to the pesticide or its metabolites in specified circumstances (e.g. daily dietary consumption over the course of a lifetime, systemic exposure each day over the course of each spraying season, year on year) that the risk assessor is confident would not be expected to produce adverse health effects in even the most sensitive individual.

The use of uncertainty factors to compensate for interspecies and intra-species variability, including that related to possible vulnerable sub-populations (e.g. infants and children), therefore addresses degrees of uncertainty in the risk assessment. There is also the important consideration of choosing the appropriate species when extrapolating to humans. Using experimental human data has been argued to reduce uncertainty in the risk assessment and to obviate partially the requirement to use uncertainty factors. However, this has raised many ethical questions, principally because of possible risks to the participants from the experimental exposure, and whether the reduced uncertainty justifies the deliberate exposure of humans to non-therapeutic agents [12].

In the context of increasing global attention to ethical oversight of biomedical research [21-23], this paper sets out to explore the ethical issues involved in such studies. The complex and inter-related scientific and ethical questions challenge the international community of occupational health professionals to provide guidance on such matters. This is particularly important since many developing countries (and even some developed countries) rely on regulatory standards derived from the regulatory risk assessment processes in the USA and Europe without applying their own procedures.

Thus, the specific objectives of this review were to examine experience of the use of human experimental studies in regulatory risk assessment, and to identify key areas of ethical disagreement in debates on the topic, with a view to informing policy decisions. As would be expected, it is not always feasible to achieve consensus; hence this review aims to highlight areas both of agreement and of disagreement, in order to foster debate on this important issue.

## Methods

Scope: For this review, experimental exposure was defined as intentional exposure of a study participant to one or more pesticides, to an extent that would not have occurred had the individual not participated in a study. The review excluded observational studies in which the investigator did not attempt to modify subjects' exposures in any way, experimental studies in which the intervention was aimed only at reducing exposures to pesticides, and experiments in which non-toxic marker substances were used as a proxy for pesticides (e.g. to assess levels and determinants of exposure in spraying operations). The review was further limited to studies in which there was no therapeutic or prophylactic objective (e.g. excluding studies to evaluate interventions aimed at controlling vectors for human diseases).

### Three approaches were used in this review

Firstly, a literature review was conducted directed at three categories of reports: a) discussion papers on human testing of pesticides; b) position papers from governments, regulatory agencies and civil society stakeholders; c) examples of studies which involved human volunteers exposed to pesticides. Searches in the peer-reviewed literature were conducted independently by three members of the workgroup and results pooled. The definition outlined above of experimental exposure to pesticides without a therapeutic or prophylactic objective served as the inclusion criterion. Keywords used in the primary search included: "pesticide/s," AND "ethics," AND "experiments" (or "experimental studies"). Secondary searches were done introducing "exploitation" as a substitute for "ethical" and "risk assessment" for "research". References cited in articles suggesting human volunteer studies were also followed up. This strategy yielded 27 empirical studies.

Additionally, position papers were sought opportunistically, through contacts and experience of Workgroup members, one of whom had been a member of the Joint FAO/WHO Meetings on Pesticide Residues (JMPR) several times. Also, studies reviewed by the JMPR were identified by two Group members through examination of its reports from 1997 to 2006. A further 10 peer reviewed papers involving experimental exposure of humans to pesticides were identified through the JMPR reports, yielding a total of 37 empirical studies involving human volunteer exposure in the peer reviewed literature.

Secondly, these 37 empirical studies involving human volunteer exposure identified from the peer-reviewed literature were reviewed for the following variables: study purpose; whether the study was explicitly addressed at pesticide authorization procedures; whether there was evidence of ethical committee approval; compounds (or groups of compounds); and main characteristics of the experimental design (route, dose - single or repeated, dose level(s), number of groups and number of subjects per group). Studies were categorized as either addressing absorption, distribution, metabolism and excretion (ADME studies); biological effects and/or toxicity; exposure; or other. It was possible to allocate a particular study to more than one category. Where data were available, the assessor was asked to comment on the statistical power of the study, on apparent compliance of the study with ethical standards and whether the study was instrumental in waiving, either totally or partially, the uncertainty factor related to interspecies extrapolation in setting reference values, therefore allowing for higher reference values than would be set based only on animal studies. Lastly, the assessor, based on his toxicological knowledge, commented on whether the study was necessary to obtain the required information, recognizing that such an evaluation required value judgments. The data abstraction tool is contained in additional file 1. Sampling for this analysis was conducted with the intention of obtaining a picture of the range of the types of studies in the published literature involving experimental exposure of humans to pesticides to inform ethical analysis rather than seeking representivity of the population of such studies.

Thirdly, an ethical analysis was applied to the arguments identified from the literature. In approaching the ethical analysis, cognizance was taken of the numerous codes providing guidance for health professionals in relation to biomedical research, particularly, the World Medical Association (WMA) Declaration of Helsinki [24], and ethical codes of the WHO-CIOMS [21] and the International Commission on Occupational Health [22].

Although the Declaration of Helsinki specifically addresses medical research, its basic principles have become the cornerstone of ethical analysis of any health research study involving human subjects. These principles include the primacy of the well-being of the research subject, the need for the research to conform to accepted scientific principles, review and approval of the study protocol by a research ethics committee, the need for an assessment of the risk to the individual and the community in comparison with the expected benefits, and free consent of the research subject on the basis of full information about these risks and benefits of participation.

## Review Findings

We begin by summarising the current regulatory positions of the USEPA, the JMPR and the EU. We next present the results of our review of empirical studies that have involved deliberate human exposure to pesticides, examining the uses which have been made of human experimental data in risk assessment for pesticides. We then provide a general ethical analysis of the issues involved, including problems that arise in relation to use of data from experimental studies conducted in the past as opposed to those that might be undertaken in the future.

### Positions of regulatory agencies on the use of data from experimental human exposure to pesticides

#### The United State Environmental Protection Agency

In the USA, the Food Quality Protection Act of 1996 introduced the concept of an additional uncertainty factor of up to 10 into the risk assessment process for pesticides, to account for possible increased sensitivity of infants and children [25]. A number of dosing studies with volunteers were subsequently submitted to the agency with the intent of providing human data which would not require application of the normal inter-species uncertainty factor. This resulted in a protracted and intensely polarized debate. Some scientists and activist groups challenged the ethical and scientific validity of the studies, contending among other things that people should not be put at risk for the purpose of reducing the stringency of regulatory standards [3,6,8,11,12]. Other scientists and industry groups argued that the human dosing studies were needed to ensure the scientific quality and accuracy of EPA's safety evaluations and that they had been and could be conducted ethically [4,5,10,26,27]. In response to the controversy, EPA commissioned the National Academy of Sciences to advise whether and under what circumstances the Agency should consider and accept intentional human dosing studies. The National Research Council committee recommended that such studies should be conducted and considered for regulatory purposes only if all of a set of specified conditions were met that related to both scientific validity and ethical standards (see appendix below) [28]:

On the basis of this advice EPA issued a final rule in 2006, which established the framework under which data from intentional human dosing studies would be considered in future [25]. The rule details the many difficult questions with which the Agency had to struggle, and the reasons for its decision. The final rule categorically prohibited any EPA-funded research, or the consideration of other ('third party') research involving 'intentional' exposure of pregnant women and children. It also extended the Federal policy that governs protection of human subjects in research ('Common Rule') to other 'intentional' exposure studies involving non-pregnant adults intended to be submitted to EPA under the pesticide laws. Furthermore, it required protocols for such studies to be submitted to EPA before the research was started, and it established an independent Human Studies Review Board to review new research proposals and research reports intended to contribute to decision-making.

However, the EPA rule does not prohibit studies where there was no ab initio intention to use the data for regulatory risk assessment, or where the study was initially conducted outside the ambit of the US regulatory system. For historical studies submitted as part of the regulatory process, the rule indicates that the EPA will consider accepting such data "unless there is clear and convincing evidence that it was fundamentally unethical or significantly deficient with respect to the ethical standards prevailing when the research was conducted." The EPA position is therefore not to exclude human studies from consideration in the regulatory risk assessment process, but to set conditions under which such studies might be considered.

#### The Joint FAO/WHO Meeting on Pesticide Residues (JMPR)

The Joint FAO/WHO Meeting on Pesticide Residues (JMPR) has several times addressed the issue of studies in human volunteers [29-31]. It has always been recognised that "human data from accidental or deliberate poisonings, biomarker monitoring studies, epidemiology studies, volunteer studies, and clinical trials on the same or structurally similar compounds can provide useful data to help establish" reference doses [31]. In addition, human data on a pesticide "should always be evaluated even when they are not used to derive" a reference value [30]. While recognising that the use of data from human volunteers in chemical risk assessment is a controversial issue, the JMPR underlined that "the use of such data can reduce the level of uncertainty inherent in extrapolating from animal models" [31]. During its meetings the JMPR pointed out the need to consider both ethical (particularly the relevant components of the Helsinki Declaration) [29] and scientific issues, particularly involving the quality and integrity of the data and the adequacy of the documentation of methods (including statistics and control values) [28]. It was stressed that the conduct and use of studies in humans "should always be considered in the context of the overall toxicological database" [31]. For instance, ARfDs based on studies in humans should provide a sufficient margin of safety for toxicological endpoints that cannot readily be addressed by such studies (e.g. developmental toxicity or carcinogenicity). By saying this, the JMPR implied that the conduct of studies in humans is only permitted after the whole toxicological database in animals has been developed and understood. Only after the relevant toxicological effects (end-points) have been identified, could a study in human volunteers be considered for its scientific relevance as a component of its ethical appropriateness.

The scientific criteria included, besides those generally applicable to studies in animals: a) group size in relation to factors such as inter-individual variation in response and the level of change considered not to be adverse, b) reference to the IPCS Guidance for the use of chemical-specific adjustment factors [32] of a minimum group size of 56 or smaller if e.g. combining results from two or more dose levels or applying an increased uncertainty factor; c) the fact that the critical end-points identified in animal studies can be investigated appropriately in human studies, d) the relevance of sex differences in toxicity. In addition the JMPR [28] indicated that "studies that have not been performed in accordance with ethical principles but are scientifically valid should be used only if the findings indicate that acceptable human exposure is lower than the level that would be determined without the use of such a study".

#### The European Union (EU)

In Europe, the regulatory framework concerning authorisation and use of pesticides has been very recently changed with the approval of Regulation 1007/2009 of 21 October 2009 that replaces Council Directive 91/414/EEC of 15 July 1991 on the placing of plant protection products (PPP) on the market and all its subsequent amendments. The Regulation firmly states that "In relation to human health, no data collected on humans shall be used to lower the safety margins resulting from tests or studies on animals" (art. 4, point 6). Therefore, it appears that, while recognising that data on the effects of human exposure are valuable, EU policy has taken the view that their usefulness is to confirm the validity of estimations made based on extrapolation from the full toxicological database regarding target organs, dose-response relationships, and the reversibility of toxic effects [33] and to 'provide reassurance on the extrapolation process' [34] without direct effects on the definition of reference values.

This was also expressed in the latest draft guidance document on the setting and application of acceptable operator exposure levels (AOEL) that precludes the use of studies conducted in humans for the purpose of determining a human No Observed Adverse Effect Level for an active substance to derive regulatory limit values, confirming their role as solely supplementary to extrapolations from appropriate studies in laboratory model species [35]. Only if human study data suggest that humans are more sensitive (which would imply a need to set a lower AOEL value) would these data take precedence over animal data. No such provision has been made for the reverse case (i.e. human data showing decreased sensitivity, allowing a higher AOEL value), a position criticized by the European Food Safety Authority's Panel on Plant Protection products and their Residues (PPR), who have argued that data from human volunteer studies should be used to derive reference values, if the studies were ethically and scientifically valid [36]. In reaching this view, the PPR Panel noted limitations of studies conducted in humans including small sample sizes, lack of sex-specific data and the possibility of studying only selected end-points. The PPR Panel stressed that human data should be used in the context of the entire toxicological profile of the PPP under consideration.'

### Review of experimental studies involving human exposure to pesticides

#### Studies published in peer-reviewed journals

Thirty-seven (37) papers, published in peer reviewed journals in the period 1947 - 2006 that met the inclusion criteria [37-73], were evaluated (See Table 1). The compounds or chemical groups addressed were: organophosphate compounds (18 papers), phenoxy herbicides (4 papers), phenol derivative fungicides (3 papers), pyrethrins or pyrethroids and piperonyl butoxide (4 papers), atrazine, captan, diethylphenylacetamide and analogs, lindane and warfarin (one paper each). Seventeen of these papers were published in the decade 1990-1999, fifteen before 1990 and six since 2000. The most common aim was to assess the ADME of one or more pesticides (28 studies) and less commonly biological effects and/or toxicity (14 studies), mostly before 1990, or exposure levels (4 studies). Three studies involved other aims (e.g. estimating exposure via foliar residues; evaluating effects specific to particular anatomic sites, and studying the interaction between two active ingredients), and 8 of the 37 studies had more than one aim. None of the papers explicitly addressed or stated an intention to address authorization or regulatory issues, although at least two, and perhaps a third study, did contribute to the setting of reference values by indicating a need to adjust an uncertainty factor. The two studies contributing directly to setting reference values included a study published in 1977 involving 5 participants exposed to 2.4D [65] and a study involving 5 participants per group exposed to mevinphos for 30 days [63].

**Table 1:** A profile of 37 human volunteer studies in the peer-reviewed literature

Authors	Study type*	Compound/Chemical Group	Route	Statistical Power	Details on Ethics Committee Approval	Higher Reference
1. Bartels et al, 1998 [37]	A	Phenols	Dermal	n.a.	Yes	No
2. Buchholz et al, 1999 [38]	A	Atrazine	Dermal	Weak	Yes	No
3. Cnubben et al, 2002 [39]		Phenols	Dermal and Intravenous	n.a.	Yes	No
4. Dick et al, 1997 [46]	A	OC	Dermal	Acceptable	Yes	No
5. DuBois and Mangun, 1947 [47]	T	OP	Oral	Weak	Not provided	No
6. Edson et al, 1967 [48]	T	OP	Oral	Acceptable	Not provided	No
7. Flannigan et al, 1985 [49]	T	Permethrin	Dermal	Acceptable	Yes	No
8. Griffin et al, 1999 [50]	A	OP	Oral and Dermal	Weak	Yes	No
9. Guthrie et al, 1976 [51]	A, O	OP	Field study	Weak	Not provided	No
10. Hayes et al, 1964 [52]	A, T, E	OP	Dermal and Ingestion	n.a.	Not provided	No
11. Kezic et, 1996 [53]	A	Dichloro-propene	Dermal	n.a.	Yes	No
12. Krieger et al, 1993 [54]	A	Captan	Dermal	Weak	Not provided	No
13. Meaklim et al, 2003 [55]	A, T	OP	Oral	Weak	Yes	Yes
14. Meuling et al 2005 [56]	A	OP	Dermal	Weak	Yes	No
15. Moeller et al, 1962 [57]	T	OP	Oral	Acceptable	Not provided	No
16. Moody et al 1992 [58]	A, E, O	Phenoxy herbicides	Oral	Weak	Yes	No
17. Moody et al, 1990 [59]	A	Phenoxy Herbicides	Dermal	Weak	Yes	No
18. Nolan et al, 1984a [60]	A, T	OP	Oral and Dermal	Weak	Not provided	No
19. Nolan et al, 1984b [61]	A	OP	Oral and Dermal	Acceptable	Yes	No
20. Ramsey et al 1992 [45]	A	Fluazifop-butyl Herbicide	Dermal	Acceptable	Yes	No
21. Rao et al, 1979 [62]	T	OP	Ingestion	Weak	Not provided	No
22. Rider et al, 1975 [63]	T	OP	Oral	Acceptable	Not provided	Yes
23. Roy et al, 2006 [40]	A, T, E	N,N-diethyl-m-toluamide and permethrin	Dermal	Weak	Yes	No
24. Sanderson and Edson, 1964 [64]	T	OP	Oral	Weak	Not provided	No
25. Sauerhoff et al, 1977 [65]	A	Phenoxy Herbicide	Oral	n.a.	Not provided	No
26. Selim et al, 1999 [66]	A	2,4-D	Oral	Weak	Not provided	Partly
27. Selim et al, 1995 [67]	A	Piperonyl butoxide	Dermal	Weak	Yes	No
28. Timchalk et al, 1998 [68]	A	Phenols	Dermal	Weak	Yes	No
29. Verberk et al, 1977 [69]	T	OP	Oral	Acceptable	Not provided	No
30. Verberk, 1977 [70]	T	OP	Oral	Acceptable	Not provided	No
31. Vessell et al, 1975 [71]	A, E, O	Warfarin (rodenticide)	Oral	Acceptable	Not provided	No
32. Wester et al, 1993 [72]	A	OP	Dermal	Acceptable	Not provided	No
33. Wester et al, 1994 [73]	A	Pyrethrin and piperonyl butoxide	Dermal	Weak	Yes	No
34. Wilks et al. 1993 [41]	A	Herbicide	Oral	Acceptable	Yes	No
35. Williams et al, 2004 [44]	A	OP	Dermal	Acceptable	Yes	No
36. Woollen et al, 1991 [43]	A	Fluazifop-butyl herbicide	Oral	Weak	Yes	No
37. Woollen et al, 1992 [42]	A	Pyrethroid Insecticide	Oral and Dermal	Acceptable	Yes	No

* A = ADME study; T = Toxicity endpoint; E = Exposure study; O = Other

In 15 papers the route of administration was dermal, in 14 oral, in four oral and dermal and in one each it was inhalation, dermal and inhalation, dermal and intravenous, dermal and oral, and oral. One of the papers described a field study. Eleven studies were based on repeated doses, and 22 on single doses. For the field study, dosing was not applicable. The number of exposure groups ranged from one to four, but one or two were the options more frequently followed. The number of subjects per group ranged from 1 to 21, but in 22 studies the number was lower than 10. The statistical power of the study was rated as acceptable in 14 cases and weak in 19 cases. In 22 cases the study was reported to have been approved by an ethics committee, whilst in the other studies the approval of an ethics committee was not requested or it was not possible to establish whether ethical approval had been sought or obtained. Nineteen studies were considered by the evaluator to comply with the Common Rule requirements, but in the remaining 15 it was not possible to answer this question. Nineteen of the evaluated studies collected information that was not obtainable in other ways, while in 7 cases the need for the study was uncertain. It should be noted that in one of the oldest studies evaluated, the experimental design was such that "severe poisonings" occurred in two of the studied subjects, in one subject on two occasions and in the second subject on three [52].

#### Studies to assess dose-response relationships for early markers of toxicity with examples

The opposing viewpoints with regard to studies undertaken for the purpose of determining a NOAEL are highlighted in an exchange of correspondence triggered by Sass and Needleman [3] who cited two studies submitted as part of the process of setting regulatory reference values in the US. In the first study [74], involving 6 exposed and 3 control subjects (all men), exposed participants ingested daily oral doses of dichlorvos at a level of approximately 0.1 mg/kg/day and had blood samples drawn every two to three days for measurement of erythrocyte cholinesterase activity. On the basis of an absence of a statistically significant difference, the authors concluded that a NOAEL could be established at 0.1 mg/kg/day. However, Sass and Needleman pointed out that there were, in fact, identified in the report, statistically significant differences in cholinesterase depression at 7, 11, 14, 16 and 18 days that were regarded by the researchers as biologically insignificant. The critique further pointed out that the lack of power in the study would invalidate any inferences for or against a NOAEL. In response, Chart and colleagues [4] disputed the biological significance of the modest cholinesterase decline since a) no clinical effects were seen and b) the decline was said to provide evidence of exposure, but was too small to indicate hazard. With regard to the power issue, Chart et al acknowledged the relatively small study size, but pointed to an overall database of hundreds of animal and human studies with dichlorvos, arguing that the available health data on any given substance should be evaluated as a whole in risk assessments.

The second study criticised by Sass and Needleman [3] was conducted by Wyld et al [75] and involved a randomized, single oral dose, double-blind placebo controlled trial of aldicarb administration, involving 24 men and 12 women, who received doses of aldicarb at three concentrations (0.01, 0.05 and 0.075 mg/kg) and a group of 22 controls (16 men and 6 women). Sass and Needleman took issue with the fact that a wide range of adverse effects (total of 24 symptoms) were reported, but that only one was considered to be treatment-related. They also pointed out that there was a significant decline in erythrocyte cholinesterase at all dose levels. They argued that the conclusion of the study that no "treatment-related clinical symptoms" were present at doses of 0.05 mg/kg or less conflicted with a previously reported Californian food poisoning incident [76], in which this dose was sufficient to necessitate hospitalisation. In response, [27] Tobia et al (2004) argued that almost half of the symptoms were in the placebo group, others were temporally unrelated, or were symptoms inconsistent with cholinesterase depression. They also disputed the NOEL of 0.05 mg/kg, arguing that the case reports cited by Goldman et al (1990) [76] contained insufficient data to derive an exposure estimate. They argued it was precisely the uncertainty over the validity of the case report data that prompted the human volunteer study, and that its findings were consistent with the rest of the aldicarb database.

#### Studies to assess human exposures when applying pesticides

Of the studies identified from the peer-reviewed published literature, only 4 were studies that evaluated exposures under experimental conditions, all of which examined dermal uptake of a pesticide. At least one study [40] was a pilot investigation that was part of a larger trial to examine interactions between various compounds used to protect armed forces against disease vectors and chemical warfare agents, and was not intended to contribute to regulatory risk assessment. None was deemed to have adequate power and none had evidence of approval by an independent ethics committee.

#### Studies to assess absorption, distribution, metabolism and excretion (ADME) of pesticides in humans

Of the studies identified for review from the peer-reviewed published literature, by far the majority (n = 28) were ADME studies and, of these, the majority (17) addressed questions that could not be answered without an experimental design. Ethical approval could be confirmed in slightly more than half of the studies (n = 22), and acceptable power calculations were evident for about half of the studies in which they were relevant for the design.

Human ADME studies have two main purposes. The first is the identification of metabolic pathways and target metabolites, which can subsequently be used in biological monitoring studies in the workplace or in the general population. Secondly, in order to interpret the results of biological monitoring studies and extrapolate back from the excretion of a metabolite to exposure to the parent compound, detailed knowledge about the human pharmacokinetics of the compound is required. The best guide to interpretation of biological monitoring studies is therefore a controlled human volunteer study, usually with a single oral dose of the compound of interest [41], although it may be desirable to study more than one dose level, and, where dermal or inhalation uptake is important, more than one route of administration. Improvements in analytical methodology enable such studies to be carried out with very low doses, improving the margins of safety for volunteers, particularly when compared to acute animal studies. ADME studies may also be important to establish whether a critical metabolic pathway occurs in humans, and thereby inform a decision about a reference value based on that pathway.

As is the case with studies of exposure, there may be circumstances in which the required information on ADME in humans can be obtained through observational studies in operators during routine use of a pesticide. However, this will not be possible for agents not yet approved for distribution, or where an experimental study is expected to generate new information that will materially enhance the risk assessment. Examples where ADME studies have contributed to improving understanding of, and opportunities to control risks, include the following:

A study of cypermethrin metabolism amongst volunteers who received a single oral and dermal dose [42] was able to demonstrate different metabolite patterns depending on the exposure route. Improved assessment of the amount absorbed across the skin in a field worker exposure study [41]was made possible by inclusion of a broader range of metabolites, illustrating that route-specific metabolism of cypermethrin, which may not have been predicted from animal studies, could be established from a human volunteer study.

A second example involved the rice herbicide molinate [41]. This study showed that the major urinary metabolite of molinate in human volunteers was very different from that found in rat studies. This striking species difference indicated that, in the absence of data from human volunteer studies, an extrapolation from rat data would underestimate the absorbed dose in field studies by a factor of 30 if the incorrect metabolite was used as a marker.

A third example of a biomarker study is one involving fluazifop-butyl (FB), a post-emergence herbicide used to control grass weeds in a large variety of broad-leaved crops. A human volunteer study to measure excretion of the acid metabolite of FB confirmed that between 80% and 93% of the dose was excreted in urine as fluazifop acid over a period of 6 days in a one-compartment model [43]. The presence of an accurate biomarker contributed to other studies' ability to characterize worker exposure and demonstrate the beneficial effects of wearing gloves during mixing and loading [45,77].

#### The JMPR database

In reviewing the JMPR database (Table 2) it was evident that experimental data involving human volunteers were more commonly used to set reference values than was the case in studies published in the peer-reviewed literature. Of the 150 active ingredients evaluated by the JMPR from 1996 to 2006, experimental data involving human volunteers were used in risk assessment for 32 of these substances. Most of the compounds were cholinesterase inhibitors (19 OPs and 3 carbamates). These studies were used to set both the ARfD and the ADI for 6 compounds and the ARfD for another 7 compounds, and, with the exception of one ADI and one ARfD, they were all cholinesterase inhibitors. In total, therefore, about 40% of those JMPR evaluations which considered experimental data from human volunteer studies used the data to set reference values.

**Table 2:** Studies involving human volunteers evaluated by the JMPR*

Compound	Group	Year	Number of toxicity studies	Number of ADME studies	Used for ADI	Used for ARfD	Comments
Carbaryl	Carb-amate	1996	3	1 (same study)	NO	NO	Carcinogenicity in mice
Dimethoate	OP	1996	1 (1967)		NO	NO	Reproductive toxicity
Mevinphos	OP	1996	2 (1975)		YES	YES	SF of 20 because N = 8
Fenthion	OP	1997	1 (1979)		YES (in 1995)	NO	Not suitable for ARfD because no effects in rats after a single much higher dose
Malathion	OP	1997	1 (1962)		NO	NO	Old study, possibly containing impurities, NOAEL in rats much higher
Methidathion	OP	1997	1 (1970)		NA	YES	SF of 10 despite N = 8 because supported by data in rats with a SF of 100
Amitraz		1998	3 (1984)	1 (1984) (same study)	NA	YES	
Dichloran		1998	1 (1962)		NO	NA	Supportive of data in dogs with a SF of 200 because of data gaps
Chlorpyrifos	OP	1999	7 (1971-1999)	1 (1999) (same study)	YES	YES	ADI based on data in animals with a SF of 100 and in humans with a SF of 10 (N = 4-6) giving the same ADI
Ethoprophos	OP	1999	1 (1986)	1 (1986, same study)	NA	NA	Study performed to assess occupational exposure
Permethrin	Pyre-throid	1999		1 (1985)	NO	NA	
Fenitrothion	OP	2000	3 (1968-1999)	1 (1999) (same study)	NO	YES	Too short duration (4 days) for setting the ADI
Imazalil	Azole	2000	1 (1979)		NO	NA	Skin irritation study
Diazinon	OP	2001	2 (1999)		NA	NO	Only in male and studies in animals showed sex difference
Methomyl	Carbamate	2001	1 (1998)		YES	YES	SF of 5 because of rapid reversibility of effects, used although N = 5 because supported by data in animals
Acephate	OP	2002	2 (1972-2000)	1 (2000) (same study)	NO	NO	Not used because brain AChE more sensitive to inhibition than RBC AChE
Ethephon		2002	3 (1971-1977)		NA	YES	
Methamidophos	OP	2002	2 (1973)		NO	NO	Studies considered supportive (giving similar ADI or ARfD)
Oxamyl	Carb-amate	2002	1 (1999)		YES	YES	Although N = 5, data in animals were supportive
Oxydemeton methyl	OP	2002	2 (1973)		NA	NO	Not used because of methodological problems
Tolylfluanid	Sulf-amide fun-gicide	2002	1 (1968)		NA	NA	Skin irritation study
Triazophos	OP	2002	4 (1971-1973)		YES	YES	N = 5-25, acceptable because performed according to the standard of the time
Malathion	OP	2003	1 (2000)	1 (2000) (same study)	NA	YES	N = 14
Paraquat		2003		1 (1984)	NO	NO	Poor skin absorption
Phospmet	OP	2003	1 (1999)		NA	YES	N = 12
Propiconazole	Azole	2004	1 (1991		NA	NA	Study for skin sensitisation
Acephate	OP	2005	2 (2000-2003)	2 (2000-2003) (same studies)	YES	YES	N = 7-14, more data on brain vs RBC AChE allowed the use of human data. Comparative metabolism data also allowed the use of a chemical specific assessment factor
Pirimiphos-methyl	OP	2006	2 (1974-1976)		NA	NO	Considered supportive since it would have given a slightly lower ARfD
Temephos	OP	2006	1 (1967)		NA	NO	Poor quality of reporting
Thiabedazole	Azole	2006	1 (1965)		NO	NO	Relevant parameters could not be assessed in humans

* Note: malathion and acephate have been evaluated twice

AChE = Acetyl Cholinesterase

ADME = Absorption, Distribution, Metabolism and Excretion

ADI = Acceptable Daily Intake

ARfD = Acute Reference Dose

NOAEL = No Observed Adverse Effect Level

RBC = Red Blood Cell

SF = Safety Factor

In the case of OPs and carbamates, the studies in volunteers have been used when there was evidence that no critical effect other than inhibition of acetylcholinesterase was observed in animals treated with the compound. For instance, because of possible carcinogenic effects of carbaryl or toxicity to reproduction of dimethoate, the ADI for these compounds was based on such effects in animals, giving a value lower than the one that would have been derived from human data. In one instance (diazinon), the data in humans were not used because the study was performed only in males and the data in animals showed a relevant sex difference in sensitivity. Some of the studies that were used to set the reference values were performed in the seventies, and although no formal GLP (good laboratory practice) standards were available at that time, these studies were considered acceptable "according to the standard" of the time.

In three studies, involving amitraz, oxamyl and methomyl, clinical signs were observed in the volunteers. In the cases of the carbamates (oxamyl and methomyl) these took the form of a moderate increase of salivation after oral treatment with the highest of the three doses used. This finding was reported as probably due to a local effect of the compounds since it was neither associated with other typical cholinergic signs nor with a level of RBC AChE inhibition that would indicate a cholinergic syndrome. In the case of amitraz, signs of neurotoxicity were observed in two subjects receiving 0.25 mg/kg bw during an ADME study. Details of the study were not available; however, it is noted that the dose was 1-2 orders of magnitude lower than that which caused neurotoxicity in several animal species, including baboons.

Seven studies included assessment of the toxicokinetics of the compound. In the case of acephate and methamidophos, the ADME data helped in reducing the uncertainty of the assessment and contributed to a partial waiving of the factors used to take account of uncertainty in setting a reference value.

It should be noted that studies in humans were generally considered of the highest value by JMPR, and generally took precedence over the data from animals. Even when the number of volunteers was relatively low (e.g.: N = 5-8), animal data were used simply to support the data in humans, rather than vice-versa.

Four studies were discarded by JMPR, either because of poor reporting, which included the impossibility of assessing the ethical standards, or because of methodological problems such as analytical methods being no longer acceptable by modern standards, or an insufficient number of subjects. These studies were generally conducted in the 1960s and '70s.

Three studies were performed to assess the potential for skin sensitisation. These studies, although not affecting the setting of reference values, may have had some impact during the authorisation processes in regulatory settings outside the JMPR.

Ten of the JMPR studies were also published in peer-reviewed journals. Four of these studies contributed to the definition of the ADI or ARfD (for chlorpyrifos and mevinphos), but only one (mevinphos) was directly used to set the value. The paper on chlorpyrifos was also available as a full study report from the company. The other studies were not used because of deficiencies of the study (n = 2), because they were performed to assess skin irritation properties (n = 1) and because they did not address toxicological end-points relevant for setting an ADI or ARfD (n = 4).

In summary, it appears that experimental studies involving human volunteers that are published in the scientific peer-reviewed literature have not been explicitly performed with the purpose of helping to set reference values. Most of the recent studies in this category deal with ADME rather than with toxic effects. However, studies submitted to the JMPR evaluation process are more likely to be directed at toxicity endpoints and less likely to appear in the peer-reviewed literature. This means that studies with toxicity endpoints appear less likely to be subject to peer review and accompanying ethical oversight (except as part of regulatory evaluation). Further, in none of the studies reviewed, in either the peer-reviewed literature or the JMPR database, was it evident that the human data contributed to a lower (more stringent) reference value than that based on animal data; rather, the converse was true - human data, if they influenced a reference value at all, led to a relaxation of the value.

## Ethical Analysis

Ethical analysis requires a structured approach to identifying all issues relevant to judgments about the research in question, particularly related to the expected and unexpected consequences and to the relative balance of burdens and benefits from participation in a research study [78-80]. Additionally, particular concerns regarding the capability of research participants, who are in a dependent position in relation to the researcher, to make informed decisions regarding their participation, would be another key issue in this analysis. Here we present a summary of an ethical analysis applied to human volunteer studies involving experimental exposure to pesticides, although a more detailed ethical analysis of the issues related to use of data from human volunteer studies is contained in Additional File 2, with detailed mapping of the issues for each stakeholder identified.

In experimental human volunteer studies, study participants are asked to carry a burden of potential adverse effects because of their exposure to the pesticide(s) under investigation without the possibility of personal benefit. The risks to participants are substantially reduced by taking into account all prior knowledge on the toxicity of the compound in question, good study design and careful monitoring of participants. But they cannot be completely eliminated with absolute certainty. The risk of toxicity will depend upon: a) the margin between the experimental dose of the pesticide and the lowest dose at which adverse effects might be expected from prior knowledge; and b) the confidence that can be placed in this prior knowledge. Whilst study design aims to minimise the risk of toxicity, there are concerns that unexpected effects may be impossible to predict [6,11,12].

Informed consent is therefore essential to protect the autonomy of participants, particularly where overt or covert pressures exist, or where scientific uncertainty about risk requires interpretation for a prospective participant whose health literacy and familiarity with scientific concepts is low. This would be aggravated in circumstances where research participants are drawn from vulnerable populations, such as inmates of institutions, mentally ill or cognitively handicapped, people with limited language proficiency (for example, migrant and seasonal workers) and even subjects who are economically or educationally disadvantaged. Moreover, informed consent would be a necessary but not sufficient requirement for ethical approval of an experimental human volunteer study, since, in the absence of a demonstrably favourable risk-benefit ratio, achieving informed consent would, of itself, not provide ethical justification for a study.

Further, payment issues complicate ethical assessment. Volunteers who take part in experimental studies of pesticides may be offered direct payment or a benefit in kind for their participation. Payments to participants in excess of their immediate direct costs (such as travel or compensation for time off work) may be viewed in many domains as undue inducements to participate [81]. At the very least, planned compensation to research subjects (monetary and otherwise, e.g. social benefits, medical examinations) must be openly reported in the oversight process required for ethical approval. In the absence of payment, participants' only reward is the possible satisfaction of having helped to advance scientific knowledge for the benefit of others.

Other parties to the research will, by definition, have different interests in the research proceeding. The study investigators may benefit through the intellectual satisfaction, publications and enhanced reputation arising from the research, as well as materially through payment. Payments, whether the source is academic institutions, public bodies or industry, may present threats to the researcher's independence if conflicts of interests are not properly managed.

The pesticide manufacturer stands to gain where a human experiment results in a risk assessment that enables its product to be registered, or re-registered or released more speedily to market. In general, a company would only commission such a study where it expected to gain financially as a long-term consequence, particularly given the high costs of mounting such studies. Users of pesticides may benefit from access to an effective means of controlling pest problems using an agent that has less uncertainty in its risk assessment, or from identification of methods for biomarker monitoring. This may have positive effects in protecting workers' health in agricultural enterprises, and in providing more options for agricultural food production and public health vector control. In theory, human volunteer studies may also help to prevent a use that would carry unacceptable risks to operators, although no empirical evidence of such an impact was found in our review.

In summary, where the study design permits the generation of meaningful data, most stakeholders other than participants will either benefit or not be adversely affected from the additional information. The critical issue, therefore, is the balance between the benefits for risk assessment from better data and the risks to the health of study participants. These competing considerations are inter-related in that the total risk will be greater if more subjects take part in a study, but the value of the information produced by the investigation will be greater with a larger sample size. This applies particularly to studies using toxicological endpoints.

A further ethical concern relates to whether pre-existing data from past human experiments, which could assist in improving risk assessment, should be used in assessing and managing the risks from pesticides, particularly in the situation where historical studies were carried out under conditions which would not be considered ethically acceptable by current standards. The benefits from use of the data may be clear, and there is no further disadvantage to the study participants since their risk of adverse effects is past. There may, however, be a societal harm, since use of data derived from unethical experiments could impinge adversely on core societal values.

Even if the study in question was considered ethically acceptable at the time it was conducted, an ethical assessment of its current use would need to take account of the full spectrum of ethical issues, including a critical assessment of both the potential benefits and any adverse impacts on human dignity. Rather than applying a general rule, such instances should be addressed on a case-by-case basis

## Discussion

The Helsinki Declaration [24] indicates clearly that "... in medical research on human subjects, considerations related to the well-being of the human subject should take precedence over the interests of science and society." At face value, the Declaration prioritises the safety and rights of study participants ahead of the interests of other stakeholders. While different articles in the Declaration have to be interpreted in the context of all other articles in the declaration, including, for example recognition that "progress in the medical field is based on research which ultimately must rest on experimentation involving human research subjects", the protection of participants from exploitation in research lies at the heart of most ethical codes. Thus, in the risk-benefit estimation, the evidence of protection of third parties from risks from which they would otherwise not be protected, or of wider preventive benefits for society, is also critical to ethical assessment.

There are consequences of experimental human volunteer studies that can be considered to confer health benefits on parties other than the study participants. For example, having data that confirm a biomarker for a new pesticide might enable future monitoring of workers or the planning of field epidemiological studies with better exposure assessment, and better understanding of the ADME for a pesticide may indicate ways to improve control of exposure, or exclude certain pathways as critical for toxic effects, thereby informing risk assessment. It would appear, however, that the quantum of benefit declines relative to the risk when toxicity endpoints such as NOAELs are the object of the study. This is particularly problematic if there is a lack of study power, a common problem in NOAEL studies, as confirmed in our review. It is therefore less obvious how reducing uncertainty in risk assessment will, of itself, lead to reduced risks to humans As would be expected given the conservative assessment factors that are applied in extrapolation from animals to humans, none of the studies reviewed in this project that have used human volunteer data, have resulted in lower exposure limits to date. They have only confirmed existing values or, in a few cases, increased reference values.

The presumption of minimal risk is key to ethical consideration. To have any confidence in 'low-doses' being well below levels of toxicity, we rely on animal toxicity data, so experimental human volunteer studies would only be feasible for chemicals with extensive animal databases. But, given the fact that we may not have all information on all possible risks, some chronic outcomes may occur at concentrations lower than those at which other recognised effects occur. For example, by analogy, there is experimental evidence that Chlorpyrifos exerts neurodevelopmental impacts at lower concentrations than would be needed to impact on cholinesterase function [82] and similar findings have been made in relation to other orgranophosphates [83-85]. While it is possible that such effects may be judged irrelevant for single dose studies in adults, it is nevertheless important that the whole existing database is taken into account when making judgments on the risk to volunteers, as elaborated further below.

Resnik and Porter (2005) [10] deal with this in relation to carcinogens by proposing that pesticides that are possible carcinogens should be excluded from any experimental human volunteer studies (although this begs the question of how a "possible carcinogen" should be defined). They also propose that the burden of proof of minimal risk be shifted onto the researcher to demonstrate that the effects sought are not serious and are reversible and suggest there should be long-term follow up of study participants to monitor for adverse outcomes. To some extent, their proposals mimic the general requirement for ethical review which must identify the appropriate balance between a credible or demonstrable benefit and the risks involved. Insistence on long-term follow-up of study participants could itself only be recommended where it was considered ethically justifiable - i.e. where there was sufficient prospect of benefit to the subject to justify the demands on their time and privacy from the follow-up process. Moreover, long-term follow up may not, of itself, be sufficient to ensure avoidance of any long-term unanticipated harms.

Furthermore, some of the debates on experimental human volunteer studies suggest that symptoms such as headache, sweating and nausea, are "not serious and are reversible" so would constitute minimal risk. But, given, for example, a view that neurobehavioural symptoms are part of a spectrum of neurotoxicity [86], such symptoms may, however, also be markers of more profound damage to the CNS and their prognostic significance would need to be evaluated as part of the risk-benefit assessment.

Lastly, much unease with the idea of permitting human experimental volunteer studies rests with the concern that this will be a slippery slope, which will, on the one hand, encourage less than ethical studies in countries where there are not established oversight structures, and, on the other hand, justify arguments for very low dosing of volunteers with other well recognised toxins and toxicants such as lead if improvements of preventive action or health protection were envisaged. This remained a disputed issue within the Workgroup's discussions.

Based on the above considerations, the workgroup could not reach a uniform position on whether experimental human volunteer studies involving pesticides should be permitted or prohibited. The issues represent complex scientific and ethical challenges and their assessment entails personal value judgements.

### Points of Agreement

However, we do propose that the following general principles may provide a starting point on which to base judgements as to the ethics of such studies.

• Uncertainty in risk assessment for pesticides is undesirable, as is harm or risk of harm to study participants. Harm or risk of harm to a study participant is less unacceptable where the participant derives a compensatory benefit in health or well-being.

• Disparities in the apportionment of harms, risks and benefits (e.g. a substantial risk and little benefit to study participants with a substantial benefit and little or no risk to other people) are undesirable.

• It must be mandatory that participants in research involving experimental exposure to pesticides give free and properly informed consent. This implies that subjects whose capacity to give informed consent is compromised should not be involved.

• Independent ethical review of experimental studies in humans must be mandatory.

We therefore propose that the following criteria must be considered in deciding whether a study involving experimental exposure of humans to pesticides can be scientifically and ethically justified:

1) Experimental studies in humans should be considered if (a) they will provide data that cannot be obtained by other methods - i.e. there is cogent evidence that there is there no other practical way to get the information needed that does not involve deliberate human exposure to pesticides; and (b) the information to be obtained is needed to address the optimal protection of health or prevention of illness; and (c) the study has been designed in a manner which ensures that its conduct, analysis and reporting will be adequate scientifically to answer the question(s) at hand.

2) Pesticides are a group of chemicals with a very large toxicological database in animals, larger than that for most other chemicals, and experimental studies in human volunteers should only be performed after consideration of all relevant toxicological and epidemiological data. Experimental exposure of humans to pesticides should not be permitted in the absence of an adequate prior toxicological database.

Further, the onus should be placed on the researcher to provide evidence based on extensive animal and, where appropriate, in vitro testing, that no adverse effects are likely in study participants.

In addition, data from experimental studies of potential adverse effects in humans should only be used to set reference values if it is clear from the toxicological database that a) the chosen endpoint is both appropriate and sensitive, and b) there are no other toxicological effects seen in animal studies for which there would be inadequate margins of exposure if the human data were used.

3) Consideration of risks relative to benefits establishes that there is minimal risk to participants relative to potential benefits from the study, and that the societal benefits of the study should substantially outweigh any expected risks to participants.

4) All of the recognized ethical standards and procedures for protecting the interests of study participants are observed, implying equitable standards of benefit-risk assessment and rigorous informed consent procedures. This should include full explanation of the current knowledge and uncertainty around the hazards of the agent/s and the risks of exposure. Payment of volunteers for their participation beyond the immediate costs that they incur could constitute an undue inducement in the context of no evident therapeutic benefit and needs careful examination.

5) While informed consent is a critical and necessary component of ethical requirements for a study, it does not alone comprise sufficient grounds to render a study ethical. Rather, in a stepwise approach to ethical assessment, the first action is to determine whether the study meets the risk-benefit threshold before considering whether the requirement for proper informed consent is also met. It would be incorrect to focus alone on questions of informed consent in these studies without a comprehensive consideration of all aspects of their ethics.

6) No studies should be conducted without prior assessment and approval by an ethics committee that is independent of both the researcher and the sponsors of the study. The review system should place the onus on the researchers to provide an ethical analysis and ethical justification for his/her study to the reviewing ethics committee as part of ethical oversight. As a check and balance on ethical conduct, this measure is critically dependent on the capacity of such ethics committees, which may vary widely between different countries and contexts, as well as the institutional environment that ensures their independence. Without properly constituted, adequately resourced and consciously independent Ethics Committees, with the requisite skills-mix for scientific and ethical assessment, this proviso may be ineffective in ensuring adequate ethical protections for studies of human exposure to pesticides. It is also particularly important that this provision apply regardless of country or region in the world, given the potential for serious global inequalities in research oversight.

7) The ethics of using findings from past studies involving experimental human exposure to pesticides should be considered on a case-by-case basis.

These above seven points of agreement concur with ethical guidelines for epidemiological studies published by CIOMS in 2009 [80].

### Points of Disagreement

Whilst agreement within the authors was possible for the above recommendations, areas of dispute that **could not be resolved **included the following:

1) Many criticisms of studies entailing experimental exposure of human volunteers to pesticides have focused on their use to reduce the need for uncertainty factors in regulatory risk assessments. The workgroup were divided over whether this was relevant to ethical analysis. One view held that use of data from experimental volunteer studies to set reference values when the studies were not appropriately designed for this purpose (and were perhaps conducted for other reasons), was a scientific problem rather than an ethical issue. It should also be taken into account that the setting of reference values is most often decided by others than the researchers involved. An alternative view was that the use of study data should always be integral to consideration of the ethics of a study at the outset, and so was not separable from ethical considerations.

2) One view regarded research intended to help relieve industry of a regulatory burden as not representing a societal but rather a private benefit, consideration of which is integral to the assessment of the relative burdens and benefits of the research. Further, research presented as having a different endpoint (e.g. an ADME study) but actually intended for the purpose of facilitating a waiver of an uncertainty factor, or discounting of a toxic effect as not relevant to humans, should not be allowed to alter reference values upward so as to avoid use of experimental human volunteer studies as a strategy to subvert stricter regulatory standards. A counter argument was that unnecessarily strict controls on one pesticide may lead to use of alternative products that in fact carry a higher risk. In this argument, provided the risks from participation in an ADME study are sufficiently small, their use for the purpose of facilitating a waiver of an uncertainty factor to allow ongoing use of a pesticide could be acceptable.

3) Related to the above, is the question of how to identify and quantify potential societal benefits from such studies. There was disagreement on the extent to which potential benefits of improved risk assessment for pesticides could be linked to potential benefits such as reducing unsafe use of pesticides, avoiding substitution by more hazardous methods of pest control, reducing hazards from vector-borne disease and improving food availability and security. The more distal the causal attribution of benefits, the more contested claims to benefits are likely to be. For example, arguing for better food availability on the basis of human testing presupposed a very long set of assumptions, which stray very far from the science and ethics of human testing studies.

4) The group also differed with regard to the nature of benefits related to data to inform more cost-effective regulation, or reducing the costs of regulation. One view held that, unless they demonstrated a need for protective measures which had earlier not been known, studies aimed at providing more cost-effective regulation, or simply reducing the cost of regulation, would not meet the criterion for benefit and therefore could not be justified in relation to the risks of harm from which ethical codes seek to protect participants, as has been argued elsewhere [12].

An alternative view in the workgroup was that such studies should be examined and reviewed following the principles of balancing benefits and risks, the ascertainment of an informed consent procedure and an independent assessment by an independent ethical review board or its equivalent. One formulation of such a weighing up of risks and benefits might be to say that intentional human dosing studies that are to be used only to improve the accuracy of a reference dose (RfD), and that otherwise provide no health or environmental benefit, can be justified only when there is reasonable certainty that participants will experience no adverse effects.

5) There was also disagreement related to estimations of minimal risk. One perspective took the view that if an adequate animal toxicology database on the chemical existed, the risks of long-term effects from appropriately low experimental exposures could be trusted to be negligible. Within the group, however, there was concern about how easy it was to assume such low risk, particularly with repeat exposures in some study designs.

6) Additionally, the group considered but was divided over a further criterion - whether there should be an absolute proscription on human studies involving pesticides that are genotoxic or that have effects that are not easily predictable with simple dose-response relationship, such as might be the case for some endocrine disrupting chemicals. One view argued that no studies should be permitted with such agents, or at the very least, they should be exceptional based on cogent motivation. Another view was more permissive and did not automatically exclude studies with such chemicals, but rather proposed that they should be evaluated on a case-by-case basis, again with careful consideration of the risk-benefit analysis.

7) There was also considerable discussion within the group over the question of scientific independence in relation to the source of the funding and the affiliations of the researchers, and its relevance to ethical considerations. One view was that the concept of 'full independence' was meaningless, given that no researchers are independent of their own preconceptions or the need to have their research funded. As a result, it was mistaken to distinguish between industry researchers and academics who erroneously considered themselves to be independent. A contrary view argued that epidemiologists and occupational health practitioners have well recognised ethical obligations to strive for full professional independence in research [21]. The fact that there are, in practice, threats to researcher independence should not be mistaken for a normative view of the absence of independence or one that renders all threats to independence as equivalent. For example, the presence of a direct commercial interest in the outcome of a study, through funding or employment relationships, was viewed as of a qualitatively different nature from the kinds of interests that a publicly funded researcher might hold. To say that is not to say that all research by industry or industry-funded researchers is problematic or unethical, only to recognize that there are additional ethical challenges.

8) Concerns were expressed that, in accepting data from experimental human volunteer studies, regulators were generating a market for such studies, which will create a climate which encourages cutting of ethical corners. In discussing this scenario, the group was unable to achieve a consensus in its analysis. While there was agreement that the role of all scientists involved was to be vigilant about opportunistic pressures applied to researchers, and to consistently uphold ethical requirements in research, there was disagreement as to how likely this scenario was, at least in the developed world. Moreover, within the group, there was a concern that pressure to secure ethical approval from private sector ethical review organisations would be likely to escalate. This position was not arguing that all human studies are a product of this cutting of corners, but for greater concern about this possibility.

9) Lastly, a perspective was advanced that any experimental studies performed with human volunteers for purposes of generating information to inform a reference value should be assumed to be unethical as the default position, until the researchers can provide a coherent case as to why it would be ethically justified in taking into account foreseen health benefits and risks/hazards involved. An alternative view was that such studies were not inherently different from other human experimental studies with pesticides and that the same criteria should be applied to judge their acceptability.

## Conclusion

All ethical guidelines converge on the position that any research on pesticides involving human subjects must be conducted such that the human dignity, integrity, health, autonomy and fundamental freedoms of the participants are respected and protected. The extent to which such provisions can be protected in the context of experimental studies involving human exposure to pesticides remains contested. While the workgroup found a number of points of convergence, there were also significant differences that could not be reconciled. Nonetheless, we have proposed in this manuscript various principles and criteria that may help in making the difficult ethical decisions that are required, but have also outlined some of the more intractable differences of opinion which are inevitable in matters of ethical analysis, and which we propose should form a basis for further debates and guidelines to achieve better resolution of this matter.

## Competing interests

The authors are employed by a range of institutions, but each author has committed their own personal interpretation and views in this paper. This work was conducted without any grant or contract funding. The University of Cape Town contributed a closed intranet site as a research site to assist the researchers in sharing information. Syngenta provided access to telephone conference facilities to allow periodic conference call communication.

MFW was employed by Syngenta, a manufacturer of crop protection products, until August 2009 and is still a shareholder. AM participated in the drafting and adoption of the statements of the JMPR and of the opinion of EFSA PPR panel on human volunteer studies quoted in the manuscript. DC has been chairman of the UK Advisory Committee on Pesticides and a member of the EFSA Plant protection Products and Residues Panel.

## Authors' contributions

All the authors contributed to the conception and design of the analysis, contributed to the drafting and revisions of the manuscripts and have given final approval for the version submitted for publication. Each author was primarily responsible for the first draft of a specific section of the paper and each author contributed to revisions of subsequent drafts.

## Appendix

### National Research Council recommendations regarding acceptance of data from human volunteer studies for pesticide registration [28]

• The study is necessary and scientifically valid - that is, it addresses an important regulatory question that cannot be answered with animal studies or observational studies involving human subjects and has been designed, conducted, and reported in a manner that ensures the study will be adequate scientifically to answer the question.

• The societal benefits of the study outweigh any anticipated risks to participants.

• Intentional human dosing studies that are to be used only to improve the accuracy of a reference dose (RfD), and that otherwise provide no health or environmental benefit, can be justified only when there is reasonable certainty that participants will experience no adverse effects.

• All of the recognized ethical standards and procedures for protecting the interests of study participants are observed, including **equitable **selection and recruitment of participants, informed consent, and independent review of the scientific and ethical merits of the study by an Institutional Review Board (IRB) or its foreign equivalent.

## Supplementary Material

Additional file 1 **Paper Evaluation Sheet**. This file includes the data capture tool used for reviewing experimental studies involving human exposure to pesticides. Click here for file

Additional file 2 **An ethical analysis: use of data from human volunteer studies involving exposure to pesticides**. This file includes a detailed exposition of the ethical analysis in assessing the ethical basis for experimental studies involving human exposure to pesticides. Click here for file
