# Supplementary material for: The ethics of human volunteer studies involving experimental exposure to pesticides: unanswered dilemmas
Source: Environ Health. 2010 Aug 18;9:50. doi: 10.1186/1476-069X-9-50 (PMC2931486; doi:10.1186/1476-069X-9-50)
Supplement: Additional file 1 — Paper Evaluation Sheet. This file includes the data capture tool used for reviewing experimental studies involving human exposure to pesticides. [file 1476-069X-9-50-S1.DOC]

**ICOH Scientific Committee on Rural Health (Agriculture, Pesticides and Organic Dusts)**

**Working Group on Experimental Study on Human Volunteers**

**Paper Evaluation Sheet**

**(prepared by Claudio Colosio and Angelo Moretto, after the CC of August 3^rd^ 2007)**

**Compiled by: AM CC DC LL MW PW**

Title of the paper…………….

Author……………………….

Year of Publication………….

Journal……………………….

Peer Reviewed Journal Yes No Not Known

“Grey literature”

Aim of the study (multiple options possible):

- ADME
- Biological effects and/or toxicity (define in a glossary)
- Exposure
- Other (please specify)……………………………….

Explicitly addressed at pesticide authorization procedures: Y N NK

Compound(s) or chemical group of the compound (?):

If applicable

- route: Oral dermal Inhalation
- single dose repeated dose (how many)
- Dose levels (number of groups):

number of subjects involved per group……………………..

Formal (power) statistical consideration ………………

Ethical Committee approval: Y N NK

Compliant with ethical rules Y N NK

Did the study allow the use of a lower uncertainty (safety, assessment) factor and the setting of a lower reference value (ethically elevant) ? Y N Not Applicable

Did the study led to the setting of a higher reference value than one possibly based on animal studies? Y N

Was the study necessary to collect the requested information Y N Doubt

Short comment of the reviewer (if any)………………………………………………………..

# GLOSSARY

- **ADME**: refers to studies aimed at identifying the key metabolic steps/metabolites or kinetic parameters in humans to be compared with those in animals. Similarities or differences between humans and animals may either increase or decrease the uncertainty of the extrapolation of toxicological effects and dose levels to humans. These include, among others, dermal penetration studies, identification of metabolic pathways (qualitative and quantitative), urinary excretion.
- Biological effects and/or toxicity: studies in humans should be conducted at dose levels not expected to cause clinical signs of toxicity. Rather changes of biochemical or physiological parameters without clinical consequences are evaluated. For instance inhibition of plasma or RBC (acetyl)cholinesterase or formation of methahemoglobin can be detected before they reach levels with clinically significant consequences.
- **Exposure**: observational studies or experimental studies can provide information on the extent of exposure in given operational conditions. These might be conducted with the active compounds, and hence biological and/or physiological parameters can also be measured, or with surrogates (e.g. dyes or fluorescent compounds) providing only data on external exposure.
