# Supplementary material for: The ethics of human volunteer studies involving experimental exposure to pesticides: unanswered dilemmas
Source: Environ Health. 2010 Aug 18;9:50. doi: 10.1186/1476-069X-9-50 (PMC2931486; doi:10.1186/1476-069X-9-50)
Supplement: Additional file 2 — An ethical analysis: use of data from human volunteer studies involving exposure to pesticides. This file includes a detailed exposition of the ethical analysis in assessing the ethical basis for experimental studies involving human exposure to pesticides. [file 1476-069X-9-50-S2.DOC]

**An ethical analysis: use of data from human volunteer studies involving exposure to pesticides.**

Ethical analysis requires a structured approach to identifying all issues relevant to judgments about the research in question, including defining the problem addressed; identification of stakeholders; collecting pertinent facts related to all possible consequences, both expected and unexpected, and including both risks and benefits; selecting ethical values (such as beneficience, non-maleficence, respect for autonomy and attention to distributive justice and equity); and assessing the implications of the research for these ethical values. Key issues to consider include the choice of the research question and the quality of informed consent, which implies careful scrutiny of the envisaged health benefits and added value for disease prevention claimed by researchers as arising from the proposed study. Researchers are obligated to take care to avoid recruitment of participants in a dependent or powerless relationship to them, or who are incapable of providing true informed consent. Examples of persons in dependent positions, or who may otherwise be vulnerable, include children, students, employees of the research group, laboratory personnel, mentally ill persons, ethnic minorities, prison inmates, homeless persons, and people with incurable diseases. Decisions on ethical acceptability are based on these considerations and on a balanced assessment of the foreseen risks and expected benefits from the research.

The first part of the analysis examines the place of different stakeholders in relation to gains and losses as a consequence of human volunteer studies, recognising that “persons” involved in research include not only research subjects but also humans in the community or society affected by the research process. We then explore ethical questions relating to the use of pre-existing data from human experimental studies that have been carried out in the past. The discussion then reflects on different ethical arguments for and against the use of the different kinds of experimental study identified in this review.

# *3.1 Impact on stakeholders*

*Study participants:* Volunteers who take part in experimental studies of pesticides may receive direct payment or a benefit in kind for their participation. Sizeable payments or, indeed, any payments above immediate costs (such as travel or compensation for time off work) may be regarded as undue inducements to participate, as they are in the Swedish context [1996]. Ultimately, permitted compensation should not be so great that participation in a study in which a prospective participant otherwise might not have chosen to take part becomes attractive. What is considered to be an undue inducement varies by time and location and should be assessed on a case by case basis by an independent ethical review committee. This implies that all planned compensation to research subjects (monetary and otherwise, e.g. social benefits, medical examinations) must be reported in the application based upon which the ethics committee will reach its decision.

In the absence of payment, participants’ only reward normally is the possible satisfaction of having helped to advance scientific knowledge for the benefit of others. However, it is the burden from such research that is the main concern for study participants, since they are the stakeholders who have most to lose, from the point of view of potential adverse effects arising from their exposures. Beside the minor burdens posed by participation in such research, which entail a time commitment and some personal discomfort (e.g. if blood samples are collected), unexpected adverse effects are possible even where the outcome measure is only an early biochemical marker of toxicity such as reduction in acetyl cholinesterase activity. The risks to participants can be reduced by taking into account all prior knowledge on the toxicity of the compound in question, as well as by good study design and careful monitoring of participants, but they cannot be eliminated altogether. The risk of toxicity will depend upon: a) the margin between the experimental dose of the pesticide and the lowest dose at which adverse effects would be expected from prior knowledge; and b) the confidence that can be placed in this prior knowledge. Of course, there may be unexpected toxicity that many believe is impossible to predict.

In addition, proper informed consent is necessary to protect the autonomy of participants, and there is a danger that their autonomy could be compromised if there are overt or covert pressures to take part (e.g. if a company employee felt that he might receive less favourable treatment if he declined to volunteer for a study). There might be other mechanisms by which the full attainment of conditions for informed consent may be rendered difficult, for example, in the way scientific uncertainty about risk is interpreted for the prospective participant. This is particularly important where the health literacy of participants is low and where there is significant uncertainty in the risk assessment prior to commencing the study. Moreover, it is important to remember the widespread recognition amongst ethicists that, while informed consent is important, of itself, it is insufficient to guarantee an ethical study.

#### *The study investigators:* Study investigators are paid for carrying out their research, and may derive other benefits from their work such as intellectual satisfaction, publications and enhanced reputation. They do not suffer important disadvantage from undertaking human experimental studies, provided the studies are approved by a properly constituted, independent research ethics committee, and are well designed and conducted. Investigators in such studies may be paid by academic institutions, public bodies or industry, which may pose various threats to their independence.

#### *The pesticide manufacturer:* The pesticide manufacturer gains most obviously where a human experiment informs the risk assessment process in a way that enables use of its product that would not otherwise be possible, or facilitates its speedier release to the market, or enables an existing product to continue its use, thereby generating revenue. In theory, there could also be benefits if a human experiment revealed unexpected, preventable risks from a pesticide, for example, in preserved reputation and reduced claims for compensation. The main disadvantage of human experimental studies to pesticide manufacturers is their cost, particularly if looking at a range of outcomes with larger sample sizes. A manufacturer would only commission such a study where it expected to gain financially as a long-term consequence.

#### *The pesticide user:* Pesticide users, who are not also study participants, would not normally be expected to suffer any disadvantage from human experimental studies. They may benefit from access to effective means of controlling pest problems that should not impair their own health because of reduced scientific uncertainty inherent in extrapolation from studies in animals and confirmation that people are not markedly more sensitive than animals to critical toxic effects. This may allow the use of an effective product that for precautionary reasons would not otherwise be permitted, identify biomonitoring targets for protecting exposed workers and help to optimise controls on exposure. In theory, human volunteer studies may also help to prevent a use that would carry unacceptable risks to operators, although no empirical evidence of such an impact was found in our review. .

Workers, as a sub-set of users, may also benefit if their jobs depend on the economic success of an agricultural enterprise in which the pesticide is used, though it is not clear that the regulatory process can necessarily be linked to job security, since phasing out one pesticide may as easily result in employment on alternative means of pest control. However, where human volunteer studies are used inappropriately in regulatory risk assessment, a false sense of security might result that would be to the user’s disadvantage.

#### *The general public:* The public has an interest in scientifically reliable and unbiased risk assessment for pesticides. This allows optimal management of benefits and risks, so that agricultural production (e.g. of food) is efficient and of adequate quantity and quality, public health organisations have agents to control vector borne disease, and other pest problems (e.g. domestic vermin/pest control) can be controlled in the best way possible. To the extent that human experimental studies reduce the uncertainty of decisions in risk management for pesticides, they are therefore to the advantage of the general public. At the same time, the public has an interest in ensuring that regulatory risk assessment is appropriately informed by sound and ethical science, and in ensuring that individual citizens are not unfairly disadvantaged by taking part in experiments on pesticides.

In summary, where the study design permits the generation of meaningful data, most stakeholders will either benefit or not be adversely affected from the additional information. The critical issue, therefore, is the balance between the benefits for risk assessment from better data, and the potential risks to the health of study participants. These competing considerations are inter-related in that the total risk will be greater if more subjects take part in a study, but the value of the information produced by the investigation will be greater with a larger sample size. This applies particularly to studies using toxicological endpoints. Conversely, the risk will be lower if there is a stronger prior evidence base on which to assess the potential for harm to participants, but where prior assessment of toxicity is more certain, the additional knowledge about toxicity that can be expected from a new study will generally be correspondingly smaller.

*Use of pre-existing data from human experiments*

The arguments that have been reviewed so far have focused on whether there is any justification for new experimental studies of pesticides in human volunteers. A distinct but related question is whether pre-existing data from human experiments should be used in assessing and managing the risks from pesticides. This is of practical relevance because, as has already been described, a number of human experimental studies with pesticides have been carried out in the past.

If a particular study objective and human experimental design is considered ethically acceptable prospectively, there is no ethical reason why data obtained from a past study with the same design should not be used for risk assessment. More complicated is the situation in which a study carried out in the past would not be considered ethically acceptable by current standards, but has produced information that could enhance risk assessment or preventive practices.

Here, the benefits from using the data may be clear, and there is no further disadvantage to the study participants since their risk of adverse effects has already been incurred, and will not alter according to whether or not the data are used. There may, however, be a societal harm if use is made of data derived unethically, since such use could reflect adversely on a society that wishes to promote a common social identity based on respect for human dignity. A second concern (not uniformly shared within the workgroup and perhaps less likely in an environment with strong ethical oversight) is that accepting data from such historical studies might, encourage unethical research in the future. For example, a researcher could pursue an experimental study of uncertain ethical status outside of ethical oversight and peer-review in the expectation that, despite its current ethical shortcomings, if presented in future as a completed study, it might be accepted by regulatory authorities to a company’s advantage.

This points to the first criterion one might use to judge whether use of data retrospectively is ethical – consideration of whether the effect of using data from an earlier experimental study will be to the advantage of the organisation that commissioned the research. If there is no advantage to the commissioning organisation (e.g. a study conducted for a pesticide manufacturer leads to tighter restrictions on one of its products), then use of the data is unlikely to be an encouragement to future unethical research.

The second criterion to be considered is whether the study, while not ethically acceptable by current standards, would nevertheless have been considered reasonable at the time that it was carried out. For example, the protocol may have been approved by an independent ethics committee before the start of data collection. This is an entirely plausible scenario, since the value judgements that are applied by ethics committees can vary from one society to another, and within a society over time.

However, any decision to use pre-existing data from studies that were deemed ethically acceptable when conducted, would need to be within the context of institutional mechanisms that ensured this did not encourage unethical research in the future. Without such checks and balances, it would be unwise to uncritically exploit such data to optimise risk assessment for the benefit of pesticide users, the public at large, and possibly other stakeholders. Rather than applying a general rule, such instances should be addressed on a case-by-case basis.

**References**

The Swedish Medical Research Council. (1996). *Guidelines for ethical evaluation of medical research involving human subjects - the policy and organization of research ethics in Sweden.* MRC report 2 1996, Swedish Medical Research Council, Stockholm, 1999..
